# Purpura vasculaire récidivant et syndrome de Sjögren: quel lien?

**DOI:** 10.11604/pamj.2015.22.92.7830

**Published:** 2015-10-01

**Authors:** Youssef Kort, Naziha Khammassi

**Affiliations:** 1 Service de Médecine Interne, Hôpital Razi, La Manouba 2010, Faculté de médecine de Tunis, Tunisie

**Keywords:** purpura vasculaire, hypergammaglobulinémie, hypergammaglobulinémie de Waldenstrom, vascular purpura, hypergammaglobulinaemia, Waldenstrom Hypergammaglobulinemia

## Image en medicine

Le purpura hypergammaglobulinémique de Waldenstrom (PHGGW) est un syndrome caractérisé par un purpura vasculaire récidivant associé à une hypergammaglobulinémie. Il peut être isolé ou plus fréquemment associé à une hémopathie maligne ou à une connectivite essentiellement le syndrome de Gougerot Sjögren (SGS). Patiente de 48 ans suivie depuis 2 ans pour un SGS associé à un lupus érythémateux systémique, qui présentait depuis environ 6 mois une éruption cutanée paroxystique et récidivante des membres inférieurs s'aggravant à l'effort. Il ne s'y associait pas de fièvre, de sueurs ou d'altération de l’état général. A l'examen cutané, il existait aux membres inférieurs des lésions érythémateuses, pétéchiales, infiltrées et ne s'effaçant pas à la vitropression évocatrices de purpura vasculaire. Le reste de l'examen était sans anomalies. A la biologie, il existait une lymphopénie à 1280/mm3, une élévation de la vitesse de sédimentation à 112 mm H1 et une hyperprotidémie à 108g/l en rapport avec une hypergammaglobulinémie d'allure polyclonale à 68g/l. Les valeurs de la CRP et de la fibrinémie étaient normales. Le dosage du complément sérique était normal. La cryoglobulinémie était négative ainsi que les sérologies de l'hépatite B, C, CMV et HIV. L’échographie trans-thoracique n'objectivait pas de signes d'endocardite infectieuse. Le diagnostic de PHGGW a été retenu devant l'association d'un purpura récidivant à une hypergammaglobulinémie et en l'absence d’éléments en faveur d'une autre étiologie à savoir une cause infectieuse, une cryoglobulinémie ou une vascularite secondaire. La patiente a été traitée par de la colchicine à 1mg/j avec une nette amélioration.

**Figure 1:**
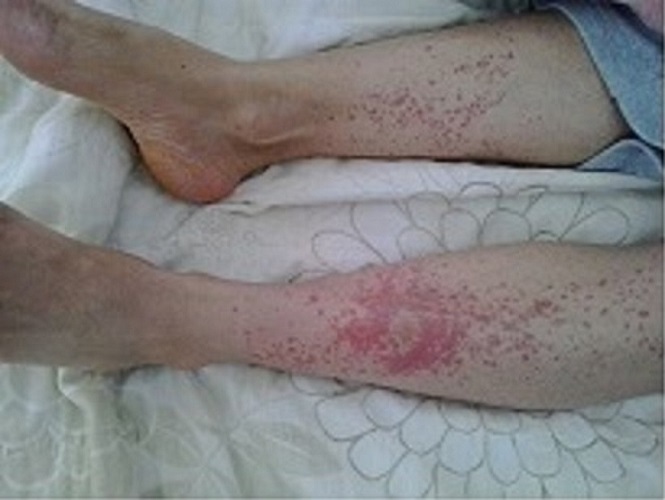
Lésions érythémateuses, pétéchiales, infiltrées aux membres inférieurs

